# The Coupled Reactance-Less Memristor Based Relaxation Oscillators for Binary Oscillator Networks

**DOI:** 10.3390/mi14020365

**Published:** 2023-01-31

**Authors:** Vladimir Rakitin, Sergey Rusakov, Sergey Ulyanov

**Affiliations:** Institute for Design Problems in Microelectronics of Russian Academy of Sciences (IPPM RAS), 124365 Moscow, Russia

**Keywords:** reactance-less memristor based oscillators, relaxation oscillators, coupled oscillators, binary oscillator networks

## Abstract

This paper discusses the application of coupled reactance-less memristor-based oscillators (MBO) with binary output signals in oscillatory networks. A class of binary-coupled memristor oscillators provides simple integration with standard CMOS logic elements. Combining MBOs with binary logic elements ensures the operation of complex information processing algorithms. The analysis of the simplest networks based on MBOs is performed. The typical reactance-less MBO with current and potential inputs is considered. The output responses for input control signals are analyzed. It is shown that the current input signal impacts primarily the rate of memristor resistance variation, while the potential input signal changes the thresholds. The exploit of the potential input for the synchronization of coupled MBOs and current control input in order to provide the necessary encoding of information is suggested. The example of the application of coupled MBOs in oscillatory networks is given, and results of simulation are presented.

## 1. Introduction

The memristor device [1,2] has successfully supplemented the modern integrated circuitry. Due to the memory property, the memristor is well suited for various information storage and processing systems, including the construction of neuromorphic systems [3,4,5,6]. Due to the inertia property of memristors, it is possible to design memristor oscillators of various types [7,8,9,10].

The properties of memristor devices open up new possibilities of constructing a relatively new class of reactance-less memristor-based oscillators (MBO) [11,12,13,14,15]. There are no standard reactive elements such as inductors and capacitors in the reactance-less MBOs. However, the active element must be present in the MBO to compensate for losses during generation when current flows through the memristor resistance. In the future, the combination of memristors with active elements can be expected [16].

The capability of memristor analog memory can be successfully used in artificial neurons (AN) and AN-based networks (ANN) [5,6,17] because they primarily meet the requirements for neuronal connections [18,19]. It can be mentioned that the memristor properties make it possible to create memristor-based AN.

It is desired to provide a certain set of properties for AN, including activation threshold, excitation, and inhibition. These properties are inherent in typical relaxation oscillators. Traditionally, such oscillators are developed using amplifiers with positive feedback or devices with negative differential resistance in combination with reactive elements. The oscillators can be represented as nonlinear dynamical systems of the first order. Their state is determined by the single variable—the phase of the periodic process. MBO, consisting of a memristor and an active element, is the simplest model of AN.

The AN models based on piecewise constant oscillators are promising [20,21]. Traditionally such oscillators were designed on standard electronic components, including amplifiers, switches, resistors, and capacitors. The piecewise constant oscillators can be characterized by transients under constant input signals, in particular, when charging or discharging capacitors with direct current. In this case, the mathematical models of AN are systems of ordinary differential equations (ODE) with constant coefficients, and the generated signals are piecewise linear functions of time. At the same time, the circuit properties inherent in complex AN models are provided, including self-excitation, braking, formation of pulses, and pulse trains.

Due to the change in memristor resistance when current flows through it, the memristor characteristics become nonlinear, which limits the construction of piecewise constant MBOs. It can be mentioned that if only a change in the sign of the current through the memristor is used, then the limitation associated with nonlinearity is removed [22]. To control such MBOs the input signal is fed not to the memristor, but to the active element. In this case, the character of the dependence of the change in the resistance of the memristor on the flowing current does not impact the output waveforms.

The simplest piecewise constant MBOs contain one memristor. In the self-excitation mode of such an oscillator, the change in the memristor resistance in time corresponds to triangular oscillations. In this case the output signal is a stationary stream of binary pulses. Synchronizability is an important property of such oscillators in their applications.

The external excitation can violate the synchronization of the oscillations of the connected MBOs. The synchronization recovery time depends on the coupling strength. This allows interconnected MBOs to modulate pulse streams to obtain the properties of oscillatory AN.

Oscillatory neural networks (ONN) based on coupled oscillators [23,24] are currently considered a promising line for solving a number of complex computational problems. An example of solving the vertex coloring optimization problem, using a computational engine based on memristor ONN, is presented in [25]. ONN can be successfully applied to solving emerging problems in the field of pattern recognition, image and audio signal processing, and robot control, as well as for the development of neuromorphic systems [26,27,28,29].

The use of binary oscillators with binary output signals [30] is a promising version for the construction of oscillatory neural networks in integrated technologies. In binary oscillator networks (BON), binary signals are exchanged and information represented by binary streams. The reactance-less MBO networks belong to this class of networks.

The rest of the paper is organized as follows. Section 2 shows the principle of construction of reactance-less MBOs. Section 3 discusses versions for coupled MBOs. An example of applying the coupled oscillators for filtering in pattern recognition tasks is given in Section 4, and a number of simulation results are presented. The drift-diffusion memristor model [2] was used for preliminary analysis of the behavior of MBOs at a qualitative level. The presented results of numerical simulation of MBOs are obtained using the model [31].

## 2. The Behavior of Reactance-Less Memristor Based Oscillator

### 2.1. Operating Principles of Oscillator

**Figure 1:**
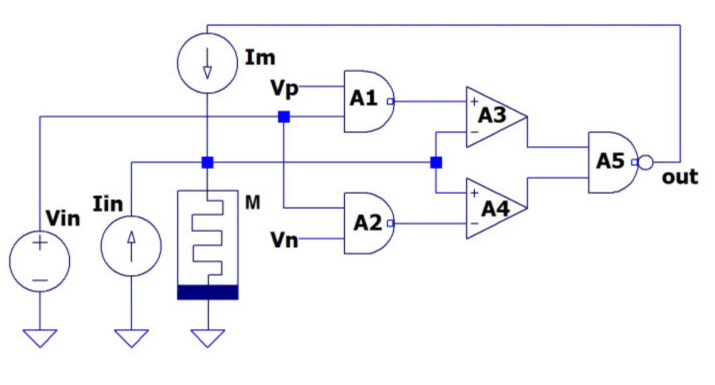
Schematic of the typical reactance-less memristor-based oscillator.

The schematic of a typical reactance-less MBO is shown in Figure 1. The circuit consists of memristor device *M*, two-threshold comparator (A3, A4), summing elements (A1, A2), and voltage control current source *Im*. The memristor is connected to the input of the comparator by anode.

The comparator converts the voltage *v* on the memristor to a binary output signal *V_out_*. The current generator *Im*, which is included in the negative feedback network of the comparator, converts the binary output signal (“0” and “1”) into negative current and positive current through the memristor (−I, +I), respectively.

The two-threshold comparator inputs are supplied with a voltage from the memristor *v* and a signal *kV_in_* that is proportional to the input signal *V_in_*. The proportionality coefficient *k* affects the coupling strength between the connected MBOs. 

The output voltage of the comparator *V_out_* is equal to the logical “0” for −*V_M_ < v < V_m_* and is equal to the logical “1” otherwise, |*V_M_|> |V_m_*|. Here *V_m_* and −*V_M_* are the threshold voltage values. The reference voltages *V_m_* and *V_M_* set the initial minimum and maximum threshold voltages, respectively. The initial threshold voltage values are set by negative *Vn* and positive *Vp* voltages on the adder in accordance with Figure 1. Here, we have *Vn =* −*V_M_, Vp = V_m_.*

As presented in Figure 1, the circuit has two inputs: *I_in_* and *V_in_*. The input current is added to the current *Im(V_out_*). The input voltage *V_in_* is subtracted from the specified values *V_m_* and *V_M_*. The current input *I_in_* is the conventional input for reactance-less MBO. 

The state of MBO is determined by the value of the memristor resistance *R(t)* and the logical state of the comparator *V_out_*. When connecting a memristor with an anode to a current generator *Im* (Figure 1), the equation

(1) dRdt=−γI,

takes place, where γ determines the switching speed of the memristor. In the framework of the drift-diffusion memristor model, this value is constant [2].

The change in the resistance of the memristor *R* is limited by the physical values of the minimal *R_on_* and maximal *R_off_* resistances. The range of variation *R* is further narrowed in the considered circuit (Figure 1) under input signal *V_in_*(*t*) due to the maximal *V_M_* and minimal *V_m_* threshold voltages of comparator:

(2) $$R_{on}<\left(V_{m}-kV_{in}\left(t\right)\right)/I=R_{m}-r_{in}\left(t\right)\leq R\left(t\right)\leq R_{M}-r_{in}\left(t\right)=\left(V_{M}-kV_{in}\left(t\right)\right)/I<R_{off}$$

Here, $R_{m}=V_{m}/I$ and $R_{M}=V_{M}/I$ are the minimal and maximal threshold resistance, respectively, and $r_{in}=kV_{in}\left(t\right)/I$ is the change in threshold resistance. 

The coupling coefficient between MBOs in the network can be introduced as input voltage sensitivity coefficient: ρ=k/I.

Thus, the generation process in MBO is reduced to the change in *R(t)* in the range of threshold resistances. In order to maintain the desired range of change in the memristor resistance and prevent it from going beyond the boundaries of the linear range, the fulfillment of the condition is additionally required as follows:

(3) $$r_{in}\left(t\right)<\min\left(R_{m}-R_{on},R_{off}-R_{M},\frac{R_{M}-R_{m}}{2}\right)$$

### 2.2. Two Control Types in MBOs

Note that external signals in MBO circuit (Figure 1) can be applied to both a memristor with an integrator function and a comparator. Usually, the integrator is controlled by current. The input signal supplied to the integrator input is then called a *current* signal. In contrast, the input signal supplied to the comparator input is called a *potential* signal. 

Below, reactance-less MBO with two possible types of input (Figure 1) are considered, and the comparison of two different MBO responses for different types of excitations is performed. Note that the state of the MBO during the generation of output pulse sequences in the presented simulation examples is determined by the character of the change in the memristor resistance.

The input signals have a different impact on the MBO oscillation process. The input current *I_in_* affects the rate of change in the resistance of the memristor, increasing the speed at the same signs of the input current and the generator current *Im,* and decreasing the speed otherwise.

The input voltage *V_in_* is applied to the comparator to change its thresholds. It determines the range of change in the memristor resistance. In this case, the threshold control principle [32] is applied.

The current and potential types of control signals (*I_in_* or *V_in_*) lead to different responses of the MBO to the shape of the input signal.

**Figure 2:**
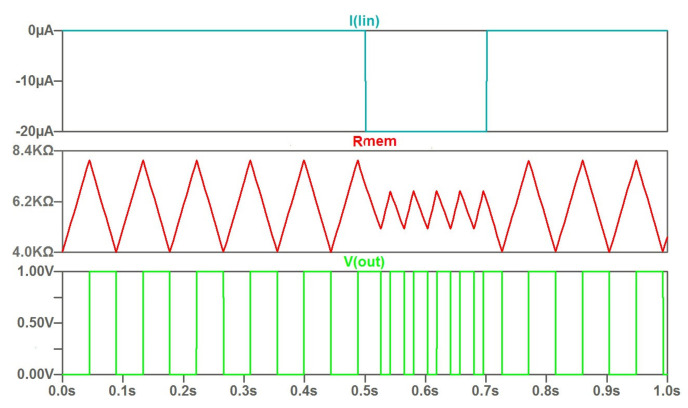
The time diagram for control by current pulse: input current pulse; character of the sawtooth change of memristor resistance *R* under current control; waveforms of voltage changes at the output of the oscillator circuit *V(out)*.

The feature of the current control consists in the variation of the rate of change of the memristor resistance and, as a consequence, the period of generated oscillations. Figure 2 illustrates this feature. The rate of varying the memristor resistance changes is by about 20% in the given example.

**Figure 3:**
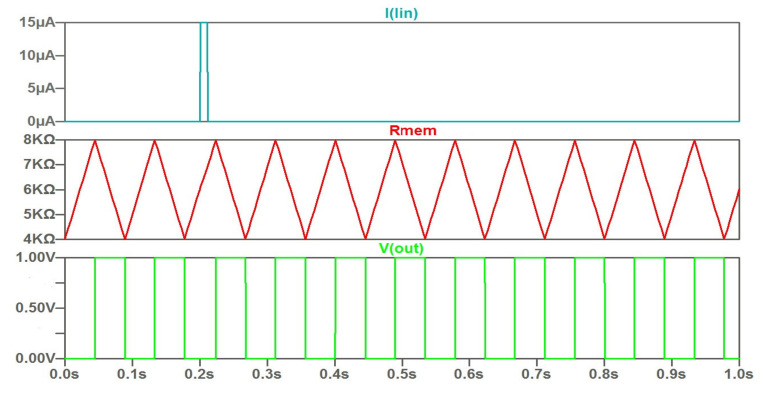
The waveforms under control by narrow current pulse of positive polarity.

**Figure 4:**
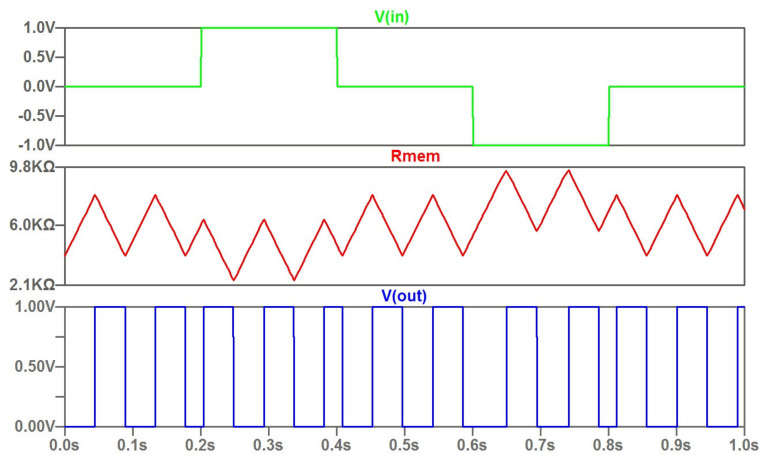
The computed waveforms of the memristor-based oscillator with controlled threshold parameters.

As a feature of the current control type, we can mark that a narrow input current pulse does not have a significant effect on the time diagram. This is shown in Figure 3. A distinctive feature of the potential control (threshold control) is shown in Figure 4. The modulation by the input signal happens at a constant rate of change of the memristor resistance. In this case, the range of variation of the memristor resistance is varied. Accordingly, the duty cycle of the output signals changes. 

**Figure 5:**
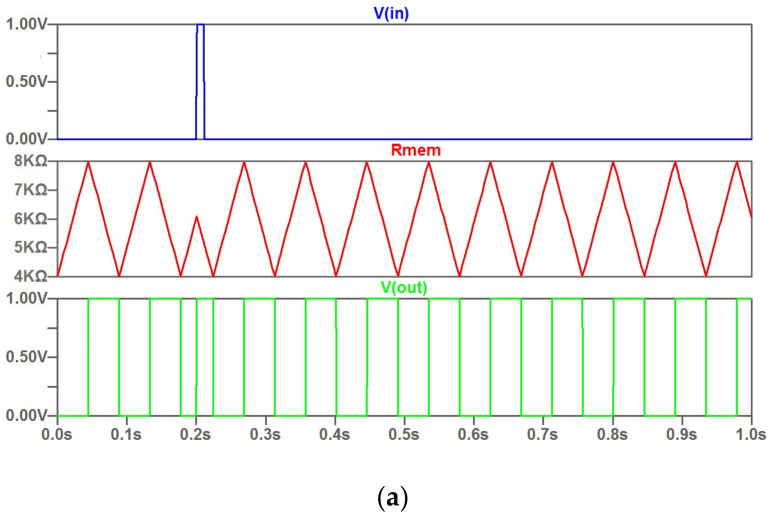

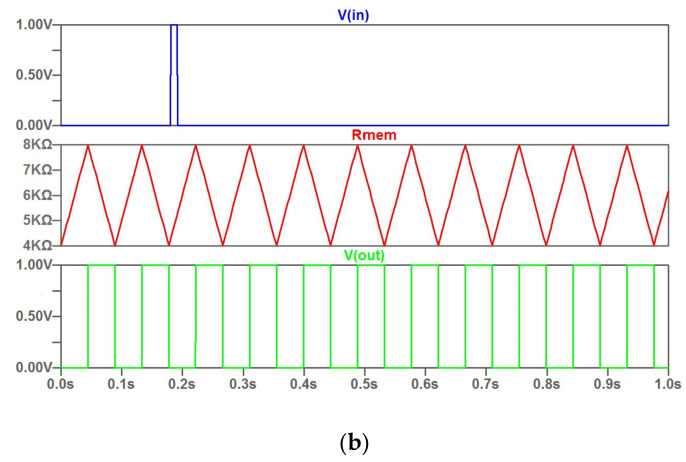
The potential control with a narrow input signal with impact (**a**) and without impact (**b**) on output pulse train.

An important functional property of the potential control that can be used is the dependence of the parameters of the output series of pulses on the arrival time of narrow input signal. Figure 5 shows examples of the moment of receipt of the input pulse on the time axis with variation.

Particular to this case, the simulation results (Figure 5A) illustrate the effect of the impact of the input pulse received in the center of the growing part of the *R (t)* sawtooth. The output pulse changes its width. In the case of an input excitation at the beginning of the rise (Figure 5B), there is no effect on the output time diagram. 

## 3. Features of Coupled Reactance-Less Memristor Based Oscillators

MBO (Figure 1) can be considered as a binary element with asynchronous behavior. A separate MBO generates a binary sequence of periodic pulses with a duty cycle of 2 in the self-excitation mode, because the switching speed of the memristor *γ* is a constant value. As part of a binary network, signals can be received at the potential input of MBO, at the current input and at both inputs. Logic elements and devices can be embedded between the output of the transmitting MBO and the input of the receiving MBO. MBOs can be also interconnected.

MBO can be used as a signal source for another receiving MBO. In this case, the transmitting MBO captures the phase of the receiving MBO. Indeed, with its output logic signal “1”, the thresholds of the receiving MBO decrease. If the receiving MBO is lagging in phase, i.e., its positive output signal MBO is late, then the maximal threshold will decrease and the phase delay will decrease. If the receiving MBO is ahead in phase, i.e., its positive output signal is generated ahead, then the minimal threshold will decrease, the transition time to the lower level will be delayed, and the advance will be reduced. The synchronization speed of the receiving MBO is proportional to the coupling coefficient *ρ*.

The rules of interaction of MBOs can be applied as follows:

- During the action of the high output level (logical “1”) of the transmitting MBO, both comparator thresholds of the receiving MBO decrease; after the completion of the action of the high output level of the transmitting MBO, the comparator thresholds of the receiving MBO are restored to their original values. The low output level (logical “0”) of the transmitting MBO does not impact on the comparator thresholds of the receiving MBO;
- Threshold changes are small enough to provide the condition of oscillations receiving MBO;
- Input potential signal does not impact the amount of current flowing through the memristor.

Each MBO in the network is in the self-excitation mode, under these conditions.

**Figure 6:**
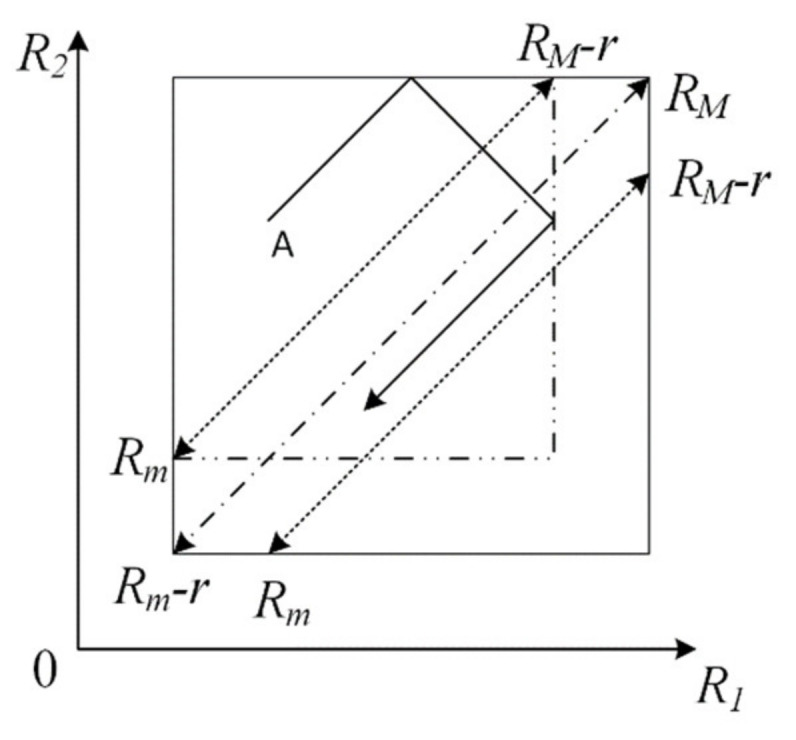
Phase plane for two coupled MBOs.

The behavior of two coupled MBOs at every time moment is described by variables *R*_1_ and *R*_2_, as well as signs of their derivatives *dR*_1_/*dt* and *dR*_2_/*dt* .Their behavior is shown on the phase plane *R*_1_ and *R*_2_ where the trajectories of the representing point are the straight lines which are parallel or perpendicular to the main diagonal of the quadrant (Figure 6). Thus, one of the four trajectories defined by the signs of the derivative *dR/dt* can pass through each point of the phase plane. 

When the boundaries of the region defined by threshold resistances are reached, the sign *dR/dt* changes, and the trajectory is mirrored from the boundary. At this point, the boundaries themselves may change.

Consider unit input signal $V_{in}\left(t\right)=1$ and define in expression $r_{in}=kV_{in}\left(t\right)/I$ the corresponding change in threshold resistance as *r*.

The region of states of coupled MBOs on the phase plane is the square with vertices (*R_M_*, *R_M_*) and $\left(R_{m}-r,R_{m}-r\right)$ lying on the main diagonal passing through these points. This square has the region of stationary trajectories of periodic motion of the system. The specific stationary trajectory characterizes the state of coupled MBOs. The region of stationary trajectories is bounded by straight lines that are parallel to the main diagonal. They cross a straight line perpendicular to the main diagonal and spaced from the vertex by the distance $r/\sqrt{2}$. This area is bounded by dotted lines in Figure 6. The stable trajectories themselves are straight parallel to the main diagonal. They correspond to synchronous oscillations. The trajectories on the main diagonal correspond to oscillations of MBO1 and MBO2 of equal amplitude.

To obtain to a stationary trajectory, it is necessary and sufficient to find an image point in the region of stationary trajectories and the same signs of derivatives. Trajectories are reflected after reaching the boundaries under initial conditions that do not meet these requirements. The segments of the trajectories of the image point with the same signs of derivatives approach the region of stationary trajectories. So, from the initial point A with positive derivatives, the image point moves to the upper boundary (Figure 6), then it is reflected from this boundary and moves to the new boundary that is the vertical line in Figure 6, passing through $R_{1}=R_{M}-r$. After reflection from it, the image point falls along stationary trajectory.

The MBO circuits can be considered as binary elements with analog memory. For MBO, the analog memory is determined by its phase relative to the reference signal of the same frequency. MBO without input signals can be used as a reference oscillator. The change of the MBO state can be provided by the current input or by the potential input, as well as by interrupting the current through the memristor. In these cases, the control signal is assumed to be binary. Thus, the MBO network can be considered an example of binary oscillator network (BON).

As a part of the BON, various digital devices can be used jointly with MBOs to control MBO behavior under external excitations. The binary signals from digital devices can be implemented into the communication circuits between MBO elements using logic elements. In fact, the BON becomes an analog-digital computing device.

## 4. Example of Application of Coupled MBOs in Oscillatory Networks 

Two types of MBO control allow different variants of their application in oscillatory computing networks. Coupled oscillators together with various additional logic elements provide a wide range of functionality, including usage in neuromorphic systems of various types.

Below is the example of the application of coupled MBOs in the task of retrieving from distorted input patterns, when the input pattern differs from the memorized patterns. The simulation results were obtained using the memristor model with window function [31]. The equivalent network and Spice subcircuit are given in this paper. The model parameters are shown in Table 1.

Some results of filtering regime [33] simulation are presented. Within frames of image recovery tasks, the relatively simple circuit example is given, taking into account the peculiarities of the considered oscillator class (Figure 1).

The frequency-shift keying (FSK) [29,34] was used in this simulation example. In the frequency shift keying, the patterns are encoded as the frequency shifts of the oscillators. FSK requires only a single stage of recognition [29]. The task involves the determination of how close the input pattern is to a class of memorized patterns [34].

The peculiarities of MBO allow the implementation of a relatively novel approach to encode the patterns using current *Im*. This became possible due to the direct connection of current *Im* with oscillator frequency.

Thus, the pattern is encoded by the values of the current generator *Im,* by, in fact, frequencies. Respectively, the encoded information can be specified by currents and can be exploited using current control type. The potential control type is used to synchronize the coupled oscillators.

In this case, the number of frequencies corresponds to the number of shades of color. In the simplest case of two colors (white pixel and black pixel), we have only two frequencies. We limit the further consideration by this partial case.

In the example under discussion, one oscillator corresponds to one neuron. There are as many pixels as there are informative oscillators in the oscillatory network.

**Figure 7:**
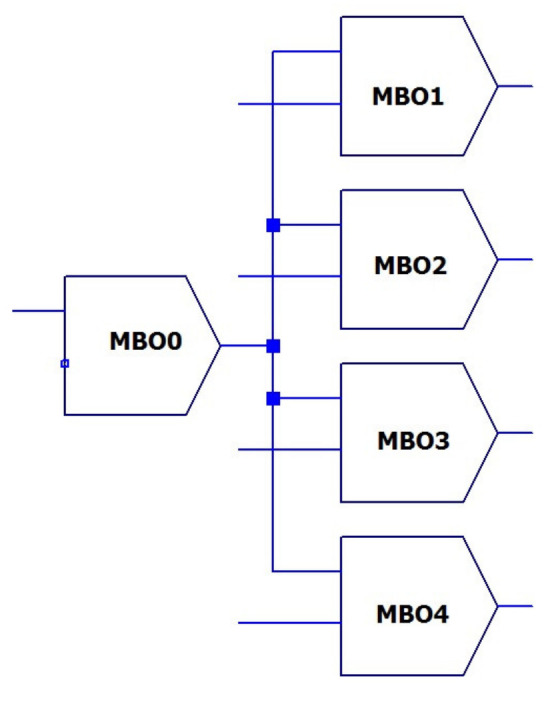
Block diagram of the coupled oscillator array.

The example of simple oscillatory network for four pixels is given in Figure 7. Each oscillator corresponds to individual pixel. Accordingly, *N* oscillators will be required for *N* pixels.

In addition, an oscillator of the reference frequency *f_0_* is included in the network. This oscillator generates some averaged (centered) frequency and operates under the control of the averaged value of the current generator *Im_0._*

The encoding of the stored pixels is performed by setting the corresponding current values of *Im_j_* .The coupling of the oscillators is assumed to be weak enough to maintain the independence of the specified frequencies *f_i_*.

Let white pixel and black pixel be encoded by the following current values: *Im_1_* = 100 uA, *Im_2_* = 200 uA. Then, the reference oscillator can be specified by the average of the current value: *Im_0_* = 150 uA.

Let the following pattern with four pixels be chosen as an example: two white pixels and two black pixels (Table 2).

**Figure 8:**
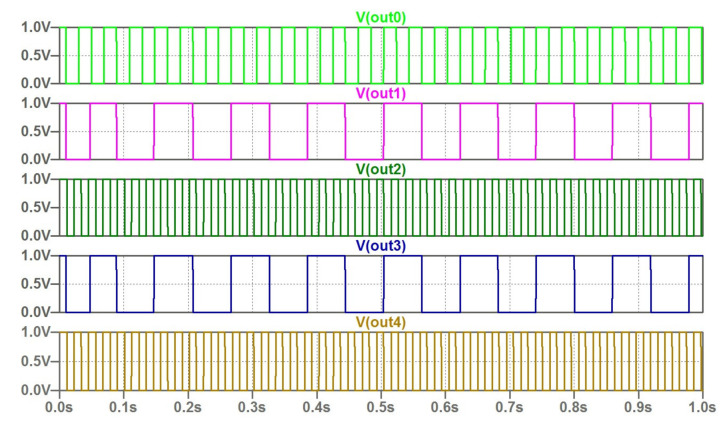
The output waveforms of the coupled oscillators according to encoded frequencies. The following set of oscillator frequencies corresponds to considered example: f_0_ = 25.2975, f_1_ = 11.2486, f_2_ = 44.6236, f_3_ = 11.2486, f_4_ = 44.6236 Hz.

The computed output waveforms for this example are given in Figure 8.

The following transformation is suggested to provide the possible synchronization of coupled oscillators for specified coupling strength in pattern recognition tasks.

(4) Imj,test>Im0Imj=Im0+θImj,test−Imj,memelseImj=Im0−θImj,test−Imj,mem

In further consideration, θ=1. Here, Imj,test is encoded input signal incoming at the current input, usually noisy input signal, and Imj,mem is memorized input signal with encoding pixels of pattern; for the given example (Table 2), the values 100 uA and 200 uA are memorized.

It can be seen that this transformation is based on the estimation of deviation Imj,test from the established value Imj,mem. The purpose of applying this transformation in practice is to organize synchronization process to retrieve the desired pattern. Due to this conversion, the deviation of the input signal value from memorized “black” or “white” encoded value is shifted to the area close to the average value corresponding to the centered frequency of the reference oscillator.

Application of the rule (4) for filtering regime in tasks of retrieving patterns is illustrated below by the simulation of a considered example of coupled oscillators.

So, if the input set of Imj,test fully corresponds to encoded values of pixels, then after conversion (4) there are no excitations at the current inputs of all the slave oscillators of network. Respectively, all the slave oscillators have the same reference frequency. The coincidence of frequencies can be considered as a sign of retrieving the “correct” pattern.

Appearance of “gray” pixel means deviation from encoded values. Application of the rule (4) leads to the corresponding deviation from the current value Im0 of the reference oscillator. Due to synchronization property of coupled oscillators, the master reference oscillator can lock the frequency of the slave oscillator. By such a way, restoring “gray” pixel of the image can be performed if this deviation lies in area of locking.

The simulation results given below confirm these considerations.

**Figure 9:**
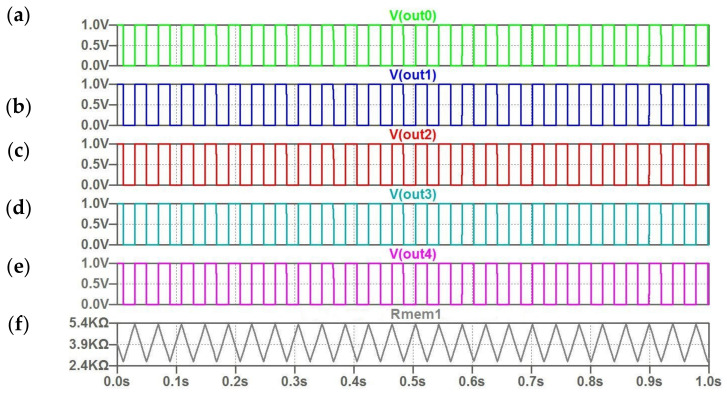
(**a**–**f**) The computed waveforms for five coupled oscillators for the “correct” image; (**f**)–the triangular character of change memristor resistance *R (t)* for the first oscillator.

Figure 9 illustrates the response of coupled oscillators on vector I_m_ set in accordance with “correct” image (Table 2). In this case, zero excitations fall on current inputs of oscillator circuits, since the real input signal is determined in this approach by the difference between stored and incoming signals. For this reason, we can see the same reference frequencies for all the oscillators. In Figure 9F, the triangular character of the memristor resistance change *R (t)* for the first oscillator is shown.

**Figure 10:**
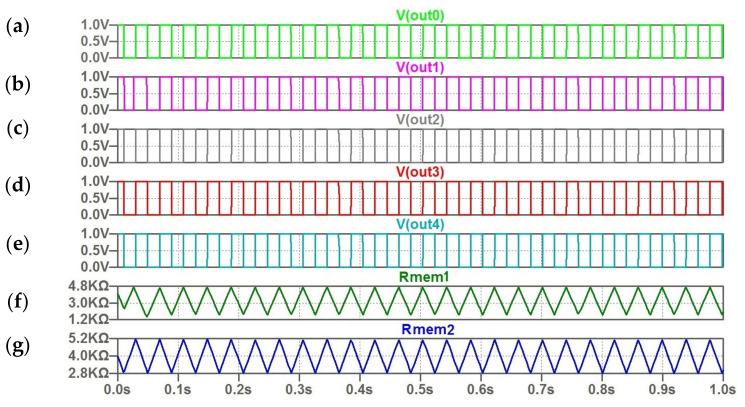
(**a**–**g**) The computed waveforms for five coupled oscillators; (**f**,**g**) the triangular character of change memristor resistances *R (t)* for the first and second oscillator.

**Figure 11:**
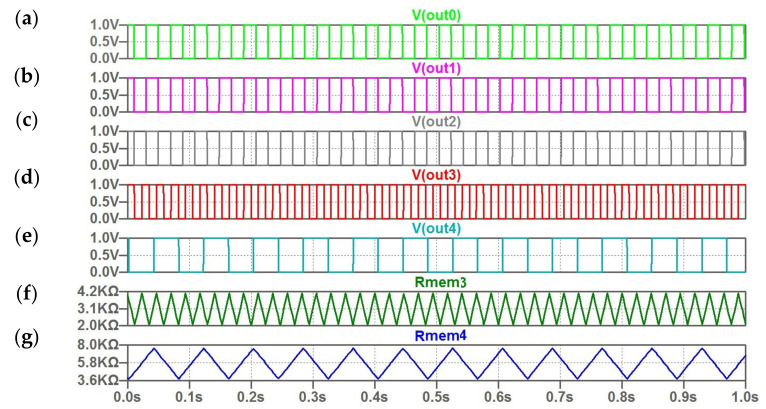
(**a**–**g**) The computed waveforms for five coupled oscillators; (**f**,**g**) the triangular character of change memristor resistances *R (t)* for the third and fourth oscillator.

The behavior of oscillator ensemble under deviation from stored encoded values is demonstrated in Figure 10 and Figure 11. For the considered example, these deviations correspond to distorted input pattern with two “gray” pixels (Table 3 and Table 4).

The waveforms in Figure 10 illustrate the version with relatively small frequency deviations (Table 3). In this case, the synchronization process is achieved and injection frequency locking is performed between reference and first oscillators and between reference and second oscillators. The speed of synchronization of oscillators is fast. The results of synchronization process can be seen in Figure 10A,C. The frequencies of first and second oscillators coincide with reference frequency. This means that the desired pattern was retrieved from distorted input pattern.

The opposite case (Table 4) is presented in Figure 11. The deviation is not suited to providing the synchronization. As we can see in Figure 11D,F, the frequencies of third and fourth oscillators are specified by individual current inputs, and they differ from the reference frequency. In this case, we can conclude regarding the lack of the desired result in the pattern retrieval process.

## 5. Conclusions

Two main control types for reactance-less relaxation memristor-based oscillators (MBO) were analyzed. The difference in the reply of reactance-less MBOs with current and potential types of input control signals was demonstrated. The signal at the current input impacts primarily the rate of memristor resistance variation. The signal at the potential input changes the thresholds without changing the rate of memristor resistance variation when switching the logical states of the MBO output.

Due to different types of control, a wide range of oscillatory circuits of various appli-cations, including binary oscillators, can be designed on the basis of the considered class of reactance-less MBOs. Two types of control provide different connections of oscillators with wide-resulting functional capabilities.

The functional capabilities of coupled oscillators on the base of MBOs, taking into account the features of synchronization modes, create prerequisites for their use in oscillatory computing networks.

The exploit of the potential input for synchronization of coupled MBOs and the provision of the necessary encoding of information with the help of current control inputs was suggested. The workability of this approach was confirmed by simulation example with the application of coupled MBOs in the task of filtering in the pattern retrieval process. The frequency shift keying was used in this simulation example, where the patterns were encoded as the frequency shifts of the oscillators.

The considered class of binary-coupled memristor oscillators provides their simple integration with standard CMOS logic elements.

## Figures and Tables

**Table 1:** The used parameters of memristor model.

Parameter	Description	Value
Ron	Resistance in ON State, [kOhm]	1
Roff	Resistance in OFF State, [kOhm]	10
Rinit	Initial resistance at t = 0, [kOhm]	4
uv	Migration coefficient, [m^2^ s^−1^ V^−1^]	10^−14^
D	Width of the thin film, [nm]	10
p	Parameter of the window function	10

**Table 2:** The example of pattern with four pixels.

White *Im_1_* = 100 uA
Black *Im_2_* = 200 uA
White *Im_3_* = 100 uA
Black *Im* *_4_* = 200 uA

**Table 3:** The example of input pattern with two gray pixels with “small” frequency deviation.

Gray *I_M1_* = 120 uA
Gray *I_M2_* = 180 uA
White *I_M3_* = 100 uA
Black *I_M4_* = 200 uA

**Table 4:** The example of input pattern with two gray pixels with “large” frequency deviation.

White *I_M1_* = 100 uA
Black *I_M2_* = 200 uA
Gray *I_M3_* = 145 uA
Gray *I_M4_* = 155 uA

## Data Availability

Not applicable.
